# Supplementary figures and images for: Development of Polyclonal Antibodies and a Serological-Based Reverse-Transcription Loop-Mediated Isothermal Amplification (S-RT-LAMP) Assay for Rice Black-Streaked Dwarf Virus Detection in Both Rice and Small Brown Planthopper
Source: Viruses. 2023 Oct 20;15(10):2127. doi: 10.3390/v15102127 (PMC10612080; doi:10.3390/v15102127)

Supplementary Figure S1

1

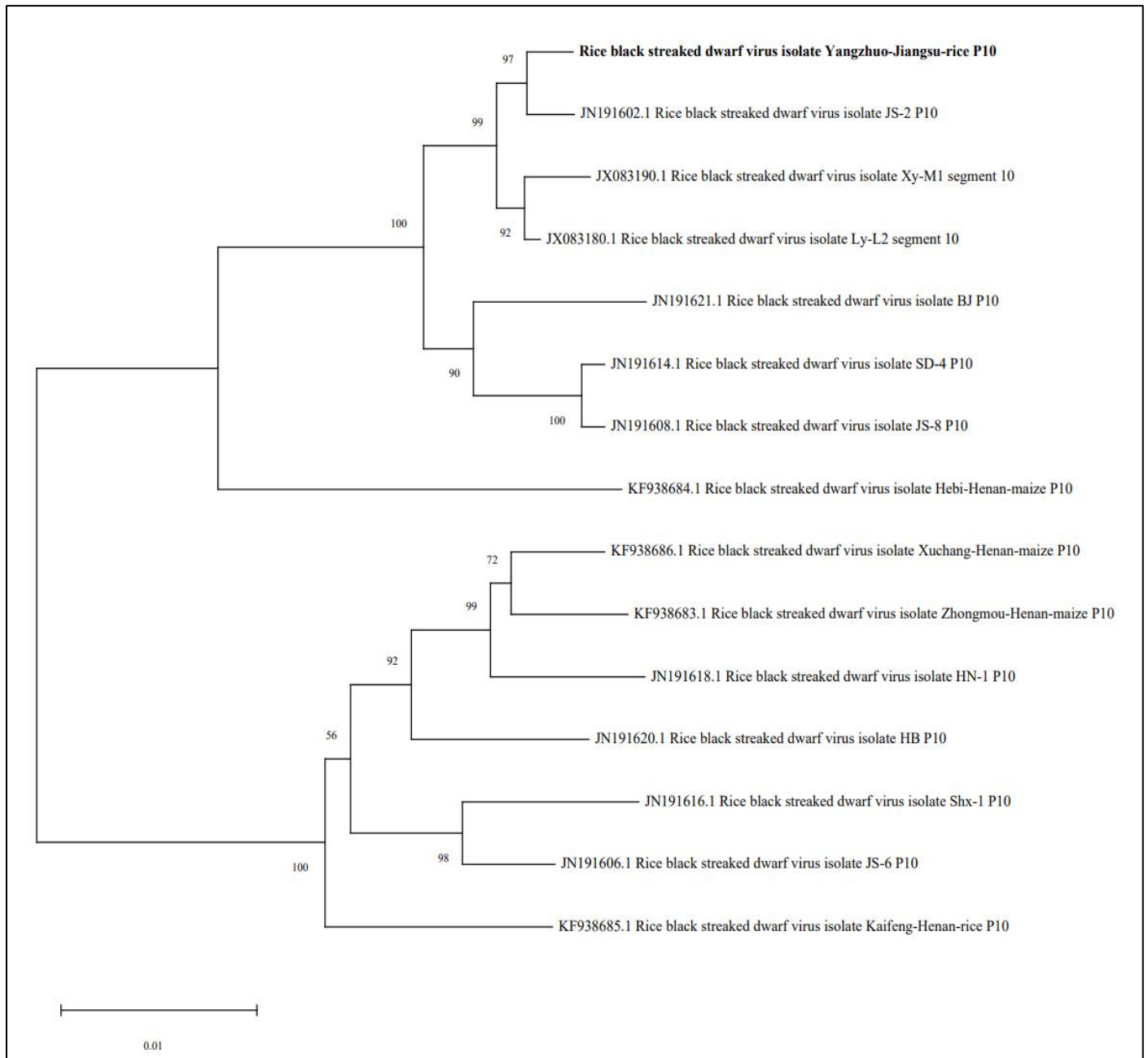

2

3

Supplement: Supplementary file 1 [file viruses-15-02127-s001.zip › viruses-2647735-supplementary.pdf]
